# Trends in the Use of Gabapentinoids and Opioids in the Postoperative Period Among Older Adults

**DOI:** 10.1001/jamanetworkopen.2023.18626

**Published:** 2023-06-16

**Authors:** Tasce Bongiovanni, Siqi Gan, Emily Finlayson, Joseph S. Ross, James D. Harrison, W. John Boscardin, Michael A. Steinman

**Affiliations:** 1 Department of Surgery, University of California, San Francisco, School of Medicine; 2 Division of Geriatrics, University of California, San Francisco, School of Medicine; 3 Northern California Institute for Research and Education, San Francisco; 4 Center for Outcomes Research and Evaluation, Yale–New Haven Hospital, New Haven, Connecticut; 5 Section of General Internal Medicine, Yale University School of Medicine, New Haven, Connecticut; 6 Department of Health Policy and Management, Yale University School of Public Health, New Haven, Connecticut; 7 Division of Hospital Medicine, University of California, San Francisco, School of Medicine; 8 Department of Medicine, University of California, San Francisco, School of Medicine; 9 Department of Epidemiology and Biostatistics, University of California, San Francisco, School of Medicine; 10 San Francisco Veterans Affairs Medical Center, San Francisco, California

## Abstract

**Importance:**

In response to the opioid epidemic, recommendations from some pain societies have encouraged surgeons to embrace multimodal pain regimens with the intent of reducing opioid use in the postoperative period, including by prescribing gabapentinoids.

**Objective:**

To describe trends in postoperative prescribing of both gabapentinoids and opioids after a variety of surgical procedures by examining nationally representative Medicare data and further understand variation by procedure.

**Design, Setting, and Participants:**

This serial cross-sectional study of gabapentinoid prescribing from January 1, 2013, through December 31, 2018, used a 20% US Medicare sample. Gabapentinoid-naive patients 66 years or older undergoing 1 of 14 common noncataract surgical procedures performed in older adults were included. Data were analyzed from April 2022 to April 2023.

**Exposure:**

One of 14 common surgical procedures in older adults.

**Main Outcomes and Measures:**

Rate of postoperative prescribing of gabapentinoids and opioids, defined as a prescription filled between 7 days before the procedure and 7 days after discharge from surgery. Additionally, concomitant prescribing of gabapentinoids and opioids in the postoperative period was assessed.

**Results:**

The total study cohort included 494 922 patients with a mean (SD) age of 73.7 (5.9) years, 53.9% of whom were women and 86.0% of whom were White. A total of 18 095 patients (3.7%) received a new gabapentinoid prescription in the postoperative period. Of those receiving a new gabapentinoid prescription, 10 956 (60.5%) were women and 15 529 (85.8%) were White. After adjusting for age, sex, race and ethnicity, and procedure type in each year, the rate of new postoperative gabapentinoid prescribing increased from 2.3% (95% CI, 2.2%-2.4%) in 2014 to 5.2% (95% CI, 5.0%-5.4%) in 2018 (*P* < .001). While there was variation between procedure types, almost all procedures saw an increase in both gabapentinoid and opioid prescribing. In this same period, opioid prescribing increased from 56% (95% CI, 55%-56%) to 59% (95% CI, 58%-60%) (*P* < .001). Concomitant prescribing also increased from 1.6% (95% CI, 1.5%-1.7%) in 2014 to 4.1% (95% CI, 4.0%-4.3%) in 2018 (*P* < .001).

**Conclusions and Relevance:**

The findings of this cross-sectional study of Medicare beneficiaries suggest that new postoperative gabapentinoid prescribing increased without a subsequent downward trend in the proportion of patients receiving postoperative opioids and a near tripling of concurrent prescribing. Closer attention needs to be paid to postoperative prescribing for older adults, especially when using multiple types of medications, which can have adverse drug events.

## Key Points

**Question:**

Is postoperative use of gabapentinoids, a medication framed as opioid sparing, increasing, and is gabapentinoid use associated with decreased opioid prescribing among older adults?

**Findings:**

In this serial cross-sectional study of postoperative pain medication prescribing for common surgical procedures in 494 922 Medicare recipients, gabapentinoid prescribing increased over a 5-year period from 2% to 5%. In this same period, opioid prescribing increased from 56% to 59%, as did concomitant prescribing.

**Meaning:**

These findings suggest that postoperative gabapentinoid prescribing for older adults is rising in addition to rather than instead of opioid use, a combination recommended to be avoided in older adults.

## Teaser

This cross-sectional study describes trends in prescribing of postoperative gabapentinoids and concomitant opioids by examining nationally representative Medicare data for multiple surgical procedures and variation by procedure type.

## Introduction

Postoperative pain management is a critical part of surgical care,^1^ and pain control is increasingly evaluated as part of quality measurement efforts. Pain control not only enhances patient satisfaction with care, but also speeds recovery and aids in patients’ ability to participate in rehabilitation. In response to the opioid epidemic and with the goal of improving pain control, surgeons have increasingly initiated multimodal pain regimens to treat pain with a goal of reducing opioid use in the postoperative period. This regimen includes a wide range of nonopioid medications, including gabapentinoids.^2^ Studies indicate that a single preoperative dose of a gabapentinoid is associated with a decrease in postoperative pain and opioid consumption at 24 hours,^3^ and some societies began to recommend uptake of multimodal pain regimens, including consideration of gabapentinoids, to decrease pain for patients undergoing surgery. Unfortunately, gabapentinoids increase postoperative sedation, dizziness, and delirium in both the short and long term,^4,5,6^ and a more recent meta-analysis^7^ suggests that perioperative gabapentinoids may not in fact decrease pain as was hoped.

Older adults (aged ≥65 years) account for almost half of all patients undergoing surgery each year in the US.^8^ In this growing patient population, gabapentinoids are often poorly tolerated because of pharmacokinetic and pharmacodynamic changes associated with aging, comorbidities, and interactions with other medications.^9^ In addition, gabapentinoids are listed in the American Geriatrics Society Beers Criteria, which explicitly detail potentially inappropriate medications for older adults, including strong recommendations to avoid using gabapentinoids with opioids except when transitioning from one to the other, and include a caution to dose renally when necessary. Concomitant use has been shown to increase risk of overdose, respiratory depression, and death, except when transitioning from one to the other.^10,11^ Additionally, postoperative prescribing of gabapentin is known to lead to prolonged use,^12^ with its own inherent risks of altered mental status and polypharmacy. Nevertheless, older age is one of several risk factors for receiving more opioids and multimodal pain medication in the postoperative period.^13,14^

The prevalence and especially the acceleration of postoperative gabapentinoid prescribing among older adults is unknown. Similarly, it is uncertain whether this shift to multimodal medications has in fact moved the needle toward its intended outcome: decreased opioid prescribing. Further, the shift toward gabapentinoid use has taken place without attention to concomitant use of opioids, a potentially dangerous combination found to lead to serious adverse events, including overdose and death.^10,11^ Therefore, we sought to describe trends in prescribing of both postoperative gabapentinoids and concomitant opioids over a recent period by examining nationally representative Medicare data for multiple surgical procedures. We further sought to understand prescribing variation by procedure type.

## Methods

This cross-sectional study followed the Strengthening the Reporting of Observational Studies in Epidemiology (STROBE) reporting guideline for cross-sectional studies (guideline responses are found in eMethods 1 in Supplement 1). The study was approved by the University of California, San Francisco, Institutional Review Board, which waived the need for informed consent based on the use of publicly available deidentified data.

### Data Source

We conducted a retrospective cross-sectional analysis of gabapentinoid prescribing by merging patient data from Medicare Carrier, Medicare Provider Analysis and Review (MedPAR), and Outpatient Files with Medicare Part D for January 1, 2013, through December 31, 2018, using a 20% Medicare sample. Medicare is a federal health insurance program that covers approximately 96% of all US citizens 65 years or older, and this data set is a representative sample of 20% of the total population. We merged Medicare Carrier, MedPAR, and Outpatient Files with Part D files and used the Master Beneficiary Summary File base file, the Chronic Condition Segment, the Other Chronic or Potentially Disabling Conditions segment, and the National Death Index segment to determine cohort composition regarding age, sex, race and ethnicity, comorbidity score, and socioeconomic disadvantage based on zip code^15^ and identify hospitalizations and fee-for-service claims at free-standing ambulatory surgical centers for specifically identified procedures, discharge location, and prescription fills. We tracked both gabapentinoid (gabapentin and pregabalin) and postoperative opioid prescribing.

### Study Population

We included patients undergoing 1 of the 14 most common noncataract surgical procedures performed in older adults^16,17^ who were 66 years or older at the time of the procedure (eTable 1 in Supplement 1) and had at least 1 prescription filled in Medicare Part D 3 months prior to surgery. To ensure we had patients who were using their Part D benefit, we only included patients with continuous Part D coverage for 3 months before and 6 months after the procedure date. For patients who had multiple procedures that met inclusion criteria over the time period, we included only their most recent procedure. We excluded patients whose discharge disposition was death or hospice,^18^ who died within 30 days after discharge, and who had 3 or more procedures on the same day. To exclude current users of gabapentinoids, we excluded patients who had any prior fill of a gabapentinoid within the 3 months prior to surgery.

We included patients 66 years or older to allow for 1 year prior to the procedure to compile comorbidities to calculate a Charlson Comorbidity Index^19^ using an updated 17-disease version more appropriate for administrative databases.^20^ The 14 surgical procedures included represent a wide range of surgical risk, anatomical regions, and specialties. We defined outpatient procedures using codes from the *Healthcare Common Procedural Coding System* and *Current Procedural Terminology* and inpatient procedures using codes from the *International Classification of Diseases, Ninth Revision, Clinical Modification*, or *ICD-10 Procedure Coding System* (eTable 2 in Supplement 1). We included specific groups of patients who had 2 different procedures on the same day if those procedures both fell into our inclusion criteria (specifically total shoulder arthroplasty with knee arthroplasty and total shoulder arthroplasty with total hip arthroplasty), and we combined the spinal procedures (laminectomy and laminotomy) into 1 category for analysis, given their extensive overlap in our data set. We defined race and ethnicity using the Research Triangle Institute race code, which is an algorithm providing an expanded definition of race and ethnicity to the Medicare data.^21^ We included race and ethnicity data to show the demographics of the cohort for generalizability to practice.

From this cohort (eFigure in Supplement 1), we analyzed patients who had a new postoperative prescription for a gabapentinoid at the time of surgery, defined as a gabapentinoid that had not been prescribed in the 3 months prior to surgery (excluding the 7 days prior to surgery).^16^ We considered a postoperative prescription as any fill between 7 days before and 7 days after the surgery (or discharge for inpatients),^12,22^ as some surgical practices prescribe medications preoperatively so that patients can have the medication ready at home. We excluded patients discharged to skilled nursing facilities (SNFs), as medication prescribing during an SNF stay cannot be ascertained with Medicare data. This excluded 17% of the entire population. We pulled the National Drug Code codes from Part D claims and then linked them with Medi-Span crosswalk files (Wolters Kluwer Health) to identify generic drug names and prescription information.

### Outcomes

The primary outcome was the rate of postoperative prescribing of gabapentinoids after surgery, defined as a prescription filled between 7 days before the procedure to 7 days after discharge from surgery, as some surgeons prescribe pain medication before the procedure so that it is already on hand when the patient is discharged, especially for outpatient procedures, as described elsewhere.^16^ We then calculated the days’ supply by using the days’ supply variable in Part D and summed all prescribing in this period. We defined which procedures most commonly had gabapentin prescribed postoperatively. We also assessed opioid prescribing (eMethods 3 in Supplement 1), as the premise of postoperative gabapentinoid use is to decrease the need for opioids. We measured opioid prescribing using the same methods we used for gabapentinoid prescribing. Additionally, we evaluated concomitant prescribing of opioids in the postoperative period, as well as oral morphine equivalents (OME) of the prescription, since concomitant use can increase the risk of adverse drug events. Oral morphine equivalents are used as a tool to compare the amount of different opioids using an equianalgesic dose chart to calculate opioid dose in a consistent and systematic way.^23^ We did not limit our population to opioid-naive patients prior to surgery, as our main focus was gabapentinoids.

### Statistical Analysis

Data were analyzed from April 2022 to April 2023. We used unpaired 2-sample *t* tests for continuous variables and χ^2^ tests for categorical variables. Statistical significance was set to 2-sided *P* < .05. To identify associations with the trends in prescribing, we constructed multivariable logistic regression models, adjusted for age, sex, race and ethnicity, and procedure type. We then constructed trends over time by analyzing the proportion of postoperative prescribing of new gabapentinoids, opioids, and mean OME across each year from 2014 to 2018, as 2013 was used to calculate comorbidities for people in the first cohort year. Trends of both gabapentinoid (gabapentin and pregabalin) and opioid prescribing during the period were analyzed using a test of linear trend in the log odds of prescribing for each year after multivariable logistic regression adjusting for procedure year, age, sex, race and ethnicity, and procedure types. Rates of gabapentinoid prescribing across years were identified using the mean estimated probability of prescription in the entire cohort for each year using the method of recycled predictions^24^; from the fitted multivariable regression model adjusting for procedure year, age, sex, race and ethnicity, and procedure types, a set of estimated probabilities were generated and then the mean calculated for each possible combination of year between 2014 and 2018 and race and ethnicity, in the full data set case mix of age, sex, and procedure type.

For trends over year by procedure groups, we ran the multivariable logistic regression with an interaction term of procedure year and procedure groups, adjusting for age, sex, and race and ethnicity. Linear trends of OME over year were analyzed by using multivariable linear regression adjusting for procedure year, age, sex, race and ethnicity, and procedure types. To analyze trends over time for procedure, we collapsed procedures into similar groups including laparoscopic, open, orthopedic, spine, and vascular (eTable 1 in Supplement 1). We conducted analyses using SAS, version 9.4 (SAS Institute Inc), Stata, version 17 (StataCorp LLC), and plots were generated with R, version 4.2.1 (R Project for Statistical Computing).

## Results

The total study cohort included 494 922 patients with a mean (SD) age of 73.7 (5.9) years; 53.9% were women and 46.1% were men. In terms of race and ethnicity, 4.9% of the patients were Black, 5.1% were Hispanic, 86.0% were White, and 4.0% were of other race or ethnicity (including American Indian or Alaska Native, Asian American or Pacific Islander, unknown, and other). A total of 18 095 patients (3.7%) received a new gabapentinoid prescription in the postoperative period (Table 1). Of those who received a new gabapentinoid prescription, 10 956 (60.5%) were women and 15 529 (85.8%) were White. There was minimal variation in the proportion of the total cohort who received a new gabapentinoid prescription by race and ethnicity (4.0% of Black patients, 3.3% of Hispanic patients, 3.6% of White patients, and 3.9% of all other groups). However, there was significant variation in prescribing by procedure type, with the most common procedure overall and the most common procedure in the new gabapentinoid prescription cohort being a total knee arthroplasty, followed by total hip arthroplasty and joint total shoulder and total knee arthroplasty (Table 2). Of the total cohort, 282 145 patients (57.0%) received a postoperative opioid prescription. Patients with a new gabapentinoid prescription received a median supply of 30 (IQR, 14-30) days.

**Table 1.** Overall Cohort for New Gabapentinoid Prescription

Characteristic	Entire cohort (N = 494 922)^a^	New gabapentinoid prescription (n = 18 095)^b^	No gabapentinoid prescription (n = 476 827)^b^	*P* value^c^
Age, mean (SD), y	73.7 (5.9)	72.7 (5.2)	73.8 (6.0)	<.001
Sex				<.001
Women	266 959 (53.9)	10 956 (4.1)	256 003 (95.9)	
Men	227 963 (46.1)	7139 (3.2)	220 824 (96.9)	
Race and ethnicity				<.01
Black	24 099 (4.9)	969 (4.0)	23 130 (96.0)	
Hispanic	25 359 (5.1)	833 (3.3)	24 526 (96.7)	
White	425 753 (86.0)	15 529 (3.6)	410 224 (96.4)	
All other^d^	19 711 (4.0)	764 (3.9)	18 947 (96.1)	
Charlson Comorbidity Index				<.001
0	200 734 (40.6)	8374 (4.2)	192 360 (95.8)	
1-2	152 011 (30.7)	5406 (3.6)	146 605 (96.4)	
3-4	78 246 (15.8)	2463 (3.1)	75 783 (96.9)	
≥5	63 931 (12.9)	1852 (2.9)	62 079 (97.1)	
Gabapentinoid supply, median (IQR), d	30 (14-30)	30 (14-30)	NA	NA
Facility type				<.001
Inpatient	301 676 (61.0)	14 516 (4.8)	287 160 (95.2)	
Outpatient	193 246 (39.0)	3579 (1.9)	189 667 (98.1)	
Inpatient LOS, median (IQR), d	2 (1-3)	2 (1-3)	2 (1-3)	<.001
Surgery planned				<.001
Yes	465 547 (94.1)	17 568 (3.8)	447 979 (96.2)	
No	29 375 (5.9)	527 (1.8)	28 848 (98.2)	
Care complexity (No. of physicians seen in prior 6 mo)				<.001
Quartile 1 (1-2)	125 520 (25.4)	5121 (4.1)	120 399 (95.9)	
Quartile 2 (3-11)	149 557 (30.2)	4430 (3.0)	145 127 (97.0)	
Quartile 3 (12-16)	97 058 (19.6)	3851 (4.0)	93 207 (96.0)	
Quartile 4 (17-161)	122 787 (24.8)	4693 (3.8)	118 094 (96.2)	

Abbreviations: LOS, length of stay; NA, not applicable.

^a^ Unless otherwise indicated, data are expressed as No. (% of column total). Percentages have been rounded and may not total 100.

^b^ Unless otherwise indicated, data are expressed as No. (% of row total). Percentages have been rounded and may not total 100.

^c^ Calculated using *t* tests for continuous variables and χ^2^ tests for categorical variables.

^d^ According to the Research Triangle Institute, this variable includes American Indian or Alaska Native, Asian or Pacific Islander, unknown, and other.

**Table 2.** Cohort by Procedure Type for New Gabapentinoid Prescription

Type of procedure	Entire cohort, No. (%)	New gabapentinoid prescription, No. (%) undergoing procedure	*P* value^a^
Knee arthroplasty	129 056 (26.1)	7730 (6.0)	<.001
Total hip arthroplasty	59 635 (12.0)	3513 (5.9)	
Total shoulder arthroplasty and total knee arthroplasty	49 604 (10.0)	366 (0.7)	
Lumbar laminotomy or lumbar laminectomy	49 132 (9.9)	2269 (4.6)	
Cholecystectomy, laparoscopic	40 199 (8.1)	1709 (4.3)	
Total shoulder arthroplasty and total hip arthroplasty	31 722 (6.4)	165 (0.5)	
Initial inguinal hernia repair, open	24 371 (4.9)	213 (0.9)	
Carotid endarterectomy	21 928 (4.4)	606 (2.8)	
Ventral hernia repair, open	21 241 (4.3)	283 (1.3)	
Total shoulder arthroplasty	20 094 (4.1)	838 (4.2)	
Initial inguinal hernia repair, laparoscopic	14 803 (3.0)	79 (0.5)	
Hysterectomy	14 357 (2.9)	109 (0.8)	
Prostatectomy, laparoscopic	12 436 (2.5)	67 (0.5)	
Low anterior resection, laparoscopic	6344 (1.3)	148 (2.3)	

^a^ Calculated using *t* tests for continuous variables and χ^2^ tests for categorical variables.

### Overall Trends

**Figure 1.**
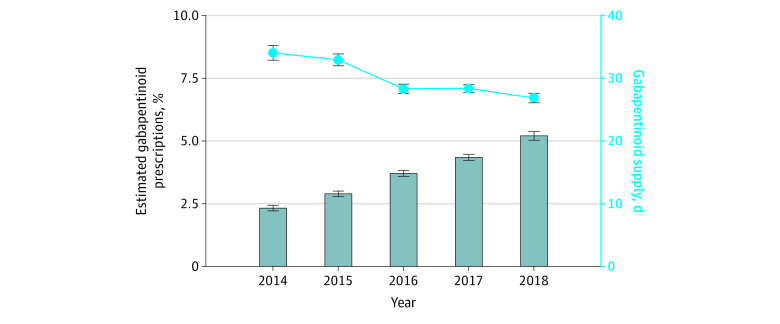
Rate of New Postoperative Gabapentinoid Prescribing Over Time Error bars indicate 95% CIs.

**Figure 2.**
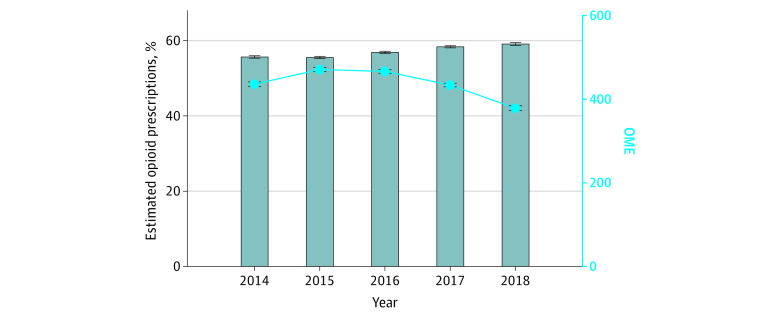
Rate of Postoperative Opioid Prescribing Over Time Error bars indicate 95% CIs. OME indicates oral morphine equivalents.

After adjusting for age, sex, race and ethnicity, and procedure type in each year, the rate of new postoperative gabapentinoid prescribing increased from 2.3% (95% CI, 2.2%-2.4%) in 2014 to 5.2% (95% CI, 5.0%-5.4%) in 2018 (*P* < .001) (Figure 1). The median supply of gabapentinoid decreased from 34 to 27 days over the entire period (*P* < .001). The rate of postoperative opioid prescribing among the total patient cohort increased from 56% (95% CI, 55%-56%) in 2014 to 59% (95% CI, 58%-60%) in 2018 (*P* < .001) (Figure 2). The rate of concomitant postoperative gabapentinoid and opioid prescribing increased from 1.6% (95% CI, 1.5%-1.7%) in 2014 to 4.1% (95% CI, 4.0%-4.3%) in 2018 (*P* < .001). The OME was more variable, starting at a mean of 436 (IQR, 431-441) in 2014, increasing in 2015 to 471 (IQR, 466-476), then trending back down to 379 (IQR, 372-384) in 2018 (*P* < .01). An OME of 436 is equivalent to fifty-eight 5-mg tablets of oxycodone and an OME of 379 is equivalent to fifty-one 5-mg tablets of oxycodone.

Trends by medication groups included increases in both opioid and gabapentinoid prescribing during the study period. There was an increase in gabapentinoid-only prescribing from 0.7% (95% CI, 0.6%-0.7%) in 2014 to 1.1% (95% CI 1.0%-1.2%) in 2018 (*P* < .001), a slight increase in opioid-only prescribing from 53.7% (95% CI, 53.3%-54%) in 2014 to 55.1% (95% CI, 54.7%-55.5%) in 2018 (*P* < .001), and an increase in concomitant opioid and gabapentinoid prescribing from 1.6% (95% CI, 1.5%-1.7%) in 2014 to 4.1% (95% CI, 4.0%-4.3%) in 2018 (*P* < .001). During the study period, patients undergoing surgery without a postoperative medication fill for an opioid and/or a gabapentinoid decreased from 43.6% (95% CI, 43.3%-43.9%) to 39.8% (95% CI, 39.4%-40.2%) in 2018 (*P* < .001).

### Trends by Procedure

**Figure 3.**
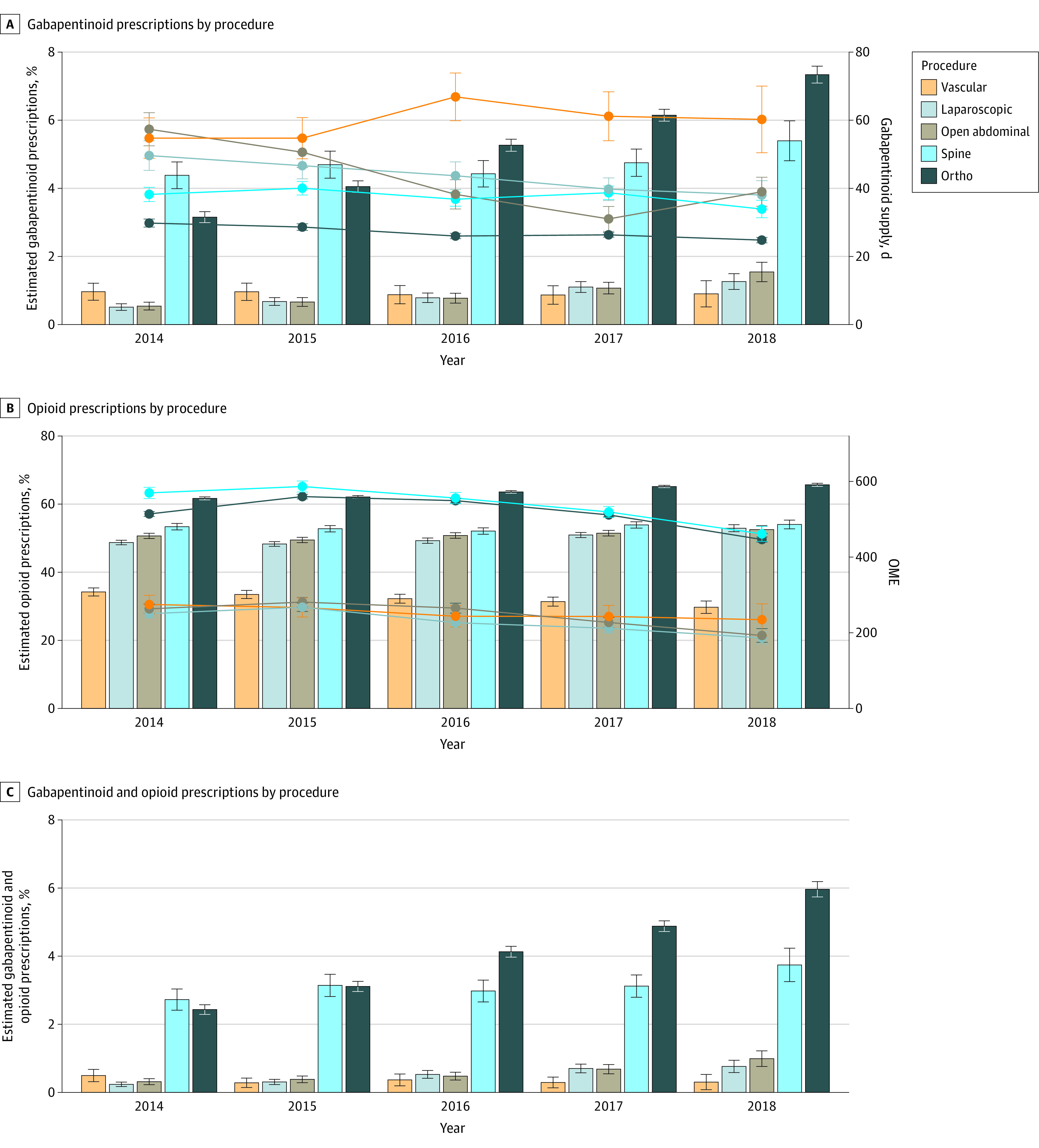
Estimated Gabapentinoid and Opioid Prescriptions by Procedure Over Time Error bars indicate 95% CIs. OME indicates oral morphine equivalents.

After adjusting for age, sex, and race and ethnicity, the rates of new postoperative gabapentinoid prescribing increased in all procedure types over time (*P* < .001) except vascular (*P* = .65) from 2014 to 2018. Spine procedures started with the highest prescription rate of 4.4% in 2014, and in 2018 the procedure group with the highest rate was orthopedic at 7.3%. The orthopedic surgery group also represented the largest change both in absolute numbers and proportionally, going from 3.2% in 2014 to 7.3% in 2018. All procedure groups except vascular also had an increase in both opioid prescribing and concomitant opioid and gabapentinoid prescribing over time (*P* < .001). There was a statistically significant decrease over time (*P* < .001) of mean OME in all groups with the exception of vascular surgery, in which mean OME decrease was not significant over time (*P* = .05) (Figure 3).

## Discussion

In this cross-sectional study of postoperative prescribing of gabapentinoids and opioids over time, we sought to describe trends in prescribing of these 2 medication groups as well as prescribing of concomitant opioids by examining nationally representative Medicare data. Overall, those who did and did not receive a new gabapentinoid prescription in the postoperative period were very similar across demographic characteristics. While many of the comparisons between the 2 cohorts were statistically significant, this likely reflects the large sample size and not necessarily a clinically meaningful difference between the groups.

Our results demonstrated a persistent increase in the prescribing of postoperative gabapentinoids over a 5-year period. Importantly, our results also reveal an increase in prescribing of postoperative opioids despite national attention to the opioid crisis and numerous studies that link opioid prescribing in the postoperative period with opioid use disorder. Finally, we also find a near tripling of concomitant gabapentinoid and opioid prescribing, all of which are statistically significant over time. The increase in the proportion of opioid prescribing over the same period persisted despite the increased use of gabapentinoids, medication meant to be opioid sparing. The increase in opioid prescribing may be balanced by the fact that OME per patient decreased; however, that decrease was only the equivalent of seven 5-mg tablets of oxycodone. These trends were reflected among patients from all racial and ethnic groups and almost all procedures.

The increase in gabapentinoid prescribing was especially notable in orthopedic procedures, while prescribing for spinal procedures started relatively high but did not increase as rapidly. Among various procedure types, the rate of change of gabapentinoid prescribing was only stable in vascular cases (carotid endarterectomy). The proportion of patients who had concomitant prescribing of gabapentinoids and opioids also rose in all procedural groups except vascular. Especially concerning is that a 2020 systematic review and meta-analysis^7^ suggests that gabapentinoids do not in fact improve postoperative pain, though 68% of the included trials studied a single dose of gabapentinoid and 71% evaluated its use in the preoperative period, not the postoperative setting.

These findings are concerning given that the prescribing of opioids in the postoperative period has been found to increase the risk of prolonged use,^25^ and the same has been described for gabapentinoid.^12^ Further, the risk of prolonged opioid use has also been found with the use of any perioperative gabapentinoid.^22^ We found that more older adults are being prescribed both of these medication types, either of which alone can be harmful, and together pose increased risks of respiratory depression and other adverse effects. Adverse events can occur for these medications, even in the setting of robust interventions to avoid them.^26^ The findings are similar to others that show increasing rates of prescribing of nonopioid medications, including muscle relaxants.^13^ Our findings suggest that the mean OME is decreasing, which may mitigate some of the danger of an increased number of patients being prescribed opioids, though that decrease may not be clinically meaningful. The proportion of opioid and OME prescribing is higher in the new gabapentinoid prescription group, suggesting that these patients are either in more pain or are receiving more pain medication than they need. In this cohort, gabapentinoid prescriptions do not appear to be reducing the proportion of patients who receive a postoperative prescription for an opioid. As has been described in prior studies,^27^ given that the risks of gabapentinoids include prolonged use, greater attention is needed to ensure they are prescribed appropriately and that concomitant use is avoided.

### Limitations

Our study has several limitations. We may have missed patients who received their supply of medications from the hospital pharmacy, which is not captured in Part D data. We excluded patients with Medicare Advantage, as diagnosis data from outpatient visits can be harder to obtain, which may limit our generalizability to fee-for-service beneficiaries. Importantly, it can be difficult to capture outpatient and diagnosis data on these patients. However, our sample size remains large despite this exclusion to allow robust assessments of the non–Medicare Advantage population. The geographic region does not necessarily represent the prescriber’s geographic location but only that of the patients. However, as these regions are quite large, they may remain a useful framework for evaluating use in larger geographic distributions. Finally, our ability to detect differences between racial and ethnic groups is limited by the manner in which Medicare traditionally categorized patients and has low validity in some groups.^28^ Because of this, certain racial and ethnic groups represented too small of a subgroup to meaningfully interpret the results and were therefore removed from the analysis by race and ethnicity. Due to availability of Medicare data, our analysis ended in 2018, so we were unable to assess whether there had been further changes in more recent years, especially given newer data on the lack of efficacy of gabapentinoids.^7^ It is possible that although gabapentinoid prescribing was rising, it is now decreasing. Additionally, we did not study single-dose preoperative or inpatient use. We did not include patients from SNFs in our analysis, therefore our results cannot be generalized to this population. Of note, while our methods of assessing medication prescribing are based on established practice, it may not perfectly capture actual medication use, as this data set was not designed to do so.

## Conclusions

The findings of this cross-sectional study suggest that new postoperative gabapentinoid prescribing is rising, without a downward trend in the proportion of patients receiving postoperative opioid prescribing. In fact, there may be an increase in postoperative opioid prescribing despite national attention on the opioid crisis. Although we found a downward trend in the OME prescribed per patient, this decrease is small. The use of gabapentinoid as opioid sparing does not appear to spare the proportion of patients receiving postoperative prescriptions for opioids from a surgical procedure, which is concerning for a medication that has its own inherent risks, especially when used concomitantly with opioids. Finally, closer attention needs to be paid to postoperative prescribing for older adults, especially when using multiple types of medications that can have adverse effects and adverse drug events.

## Notes

Supplement 1. **eTable 1.** Included Surgical Procedures and Groupings **eTable 2.** List of Outpatient Procedures Using *HCPCS* or *CPT* codes and Inpatient Procedures Using *ICD-9-CM* or *ICD-10-PCS* Codes **eFigure.** Flowchart Inclusion Criteria **eMethods 1.** STROBE and RECORD Guideline Responses **eMethods 2.** Surgical Procedures Explanation **eMethods 3.** List of Opioids Used in Analysis

Supplement 2.
Data Sharing Statement

## References

[1] Gan TJ, Lubarsky DA, Flood EM, . Patient preferences for acute pain treatment. Br J Anaesth. 2004;92(5):681-688. doi:10.1093/bja/aeh123 15003986

[2] Bongiovanni T, Anderson TS, Marcum ZA. Perioperative gabapentin use in older adults: revisiting multimodal pain management. JAMA Intern Med. 2022;182(11):1127-1128. doi:10.1001/jamainternmed.2022.3757 36121647

[3] Grover VK, Mathew PJ, Yaddanapudi S, Sehgal S. A single dose of preoperative gabapentin for pain reduction and requirement of morphine after total mastectomy and axillary dissection: randomized placebo-controlled double-blind trial. J Postgrad Med. 2009;55(4):257-260. doi:10.4103/0022-3859.58928 20083871

[4] Hurley RW, Cohen SP, Williams KA, Rowlingson AJ, Wu CL. The analgesic effects of perioperative gabapentin on postoperative pain: a meta-analysis. Reg Anesth Pain Med. 2006;31(3):237-247. doi:10.1097/00115550-200605000-00011 16701190

[5] Mishriky BM, Waldron NH, Habib AS. Impact of pregabalin on acute and persistent postoperative pain: a systematic review and meta-analysis. Br J Anaesth. 2015;114(1):10-31. doi:10.1093/bja/aeu293 25209095

[6] Park CM, Inouye SK, Marcantonio ER, . Perioperative gabapentin use and in-hospital adverse clinical events among older adults after major surgery. JAMA Intern Med. 2022;182(11):1117-1127. doi:10.1001/jamainternmed.2022.3680 36121671 PMC9486639

[7] Verret M, Lauzier F, Zarychanski R, ; Canadian Perioperative Anesthesia Clinical Trials (PACT) Group. Perioperative use of gabapentinoids for the management of postoperative acute pain: a systematic review and meta-analysis. Anesthesiology. 2020;133(2):265-279. doi:10.1097/ALN.0000000000003428 32667154

[8] Berian JR, Rosenthal RA, Baker TL, . Hospital standards to promote optimal surgical care of the older adult: a report from the Coalition for Quality in Geriatric Surgery. Ann Surg. 2018;267(2):280-290. doi:10.1097/SLA.0000000000002185 28277408

[9] McKeown JL. Pain management issues for the geriatric surgical patient. Anesthesiol Clin. 2015;33(3):563-576. doi:10.1016/j.anclin.2015.05.010 26315638

[10] 2019 American Geriatrics Society Beers Criteria Update Expert Panel. American Geriatrics Society 2019 Updated AGS Beers Criteria for potentially inappropriate medication use in older adults. J Am Geriatr Soc. 2019;67(4):674-694. 30693946 10.1111/jgs.15767

[11] Bykov K, Bateman BT, Franklin JM, Vine SM, Patorno E. Association of gabapentinoids with the risk of opioid-related adverse events in surgical patients in the United States. JAMA Netw Open. 2020;3(12):e2031647. doi:10.1001/jamanetworkopen.2020.31647 33372975 PMC7772715

[12] Bongiovanni T, Gan S, Finlayson E, . Prolonged use of newly prescribed gabapentin after surgery. J Am Geriatr Soc. 2022;70(12):3560-3569. doi:10.1111/jgs.18005 36000860 PMC9771946

[13] Soprano SE, Hennessy S, Bilker WB, Leonard CE. Assessment of physician prescribing of muscle relaxants in the United States, 2005-2016. JAMA Netw Open. 2020;3(6):e207664. doi:10.1001/jamanetworkopen.2020.7664 32579193 PMC7315288

[14] Johansen ME. Gabapentinoid use in the United States 2002 through 2015. JAMA Intern Med. 2018;178(2):292-294. doi:10.1001/jamainternmed.2017.7856 29297045 PMC5838608

[15] Kind AJH, Buckingham WR. Making neighborhood-disadvantage metrics accessible—the Neighborhood Atlas. N Engl J Med. 2018;378(26):2456-2458. doi:10.1056/NEJMp1802313 29949490 PMC6051533

[16] Thiels CA, Habermann EB, Hooten WM, Jeffery MM. Chronic use of tramadol after acute pain episode: cohort study. BMJ. 2019;365:l1849. doi:10.1136/bmj.l1849 31088782 PMC6514531

[17] Deiner S, Westlake B, Dutton RP. Patterns of surgical care and complications in elderly adults. J Am Geriatr Soc. 2014;62(5):829-835. doi:10.1111/jgs.12794 24731176 PMC4024102

[18] Zhu Y, Stearns SC. Post-acute care locations: hospital discharge destination reports vs Medicare claims. J Am Geriatr Soc. 2020;68(4):847-851. doi:10.1111/jgs.16308 31880309

[19] Charlson ME, Pompei P, Ales KL, MacKenzie CR. A new method of classifying prognostic comorbidity in longitudinal studies: development and validation. J Chronic Dis. 1987;40(5):373-383. doi:10.1016/0021-9681(87)90171-8 3558716

[20] Deyo RA, Cherkin DC, Ciol MA. Adapting a clinical comorbidity index for use with *ICD-9-CM* administrative databases. J Clin Epidemiol. 1992;45(6):613-619. doi:10.1016/0895-4356(92)90133-8 1607900

[21] Research Data Assistance Center. Research Triangle Institute (RTI) Race Code. Accessed September 20, 2022. https://resdac.org/cms-data/variables/research-triangle-institute-rti-race-code

[22] Chen C, Tighe PJ, Lo-Ciganic WH, Winterstein AG, Wei YJ. Perioperative use of gabapentinoids and risk for postoperative long-term opioid use in older adults undergoing total knee or hip arthroplasty. J Arthroplasty. 2022;37(11):2149-2157.e3. doi:10.1016/j.arth.2022.05.018 35577053 PMC9588599

[23] Oregon Pain Guidance. Opioid conversion calculator for morphine equivalents. 2023. Accessed March 1, 2023. https://www.oregonpainguidance.org/opioidmedcalculator/

[24] Graubard BI, Korn EL. Predictive margins with survey data. Biometrics. 1999;55(2):652-659. doi:10.1111/j.0006-341X.1999.00652.x 11318229

[25] Brummett CM, Waljee JF, Goesling J, . New Persistent opioid use after minor and major surgical procedures in US adults. JAMA Surg. 2017;152(6):e170504. doi:10.1001/jamasurg.2017.0504 28403427 PMC7050825

[26] Bongiovanni T, Steinman MA. Adverse drug events after hospitalization—are we missing the mark? JAMA Intern Med. 2021;181(5):618-619. doi:10.1001/jamainternmed.2020.9282 33646266 PMC9258471

[27] Billig JI, Sears ED, Gunaseelan V, . Inappropriate preoperative gabapentinoid use among patients with carpal tunnel syndrome. J Hand Surg Am. 2020;45(8):677-689.e5. doi:10.1016/j.jhsa.2020.04.011 32487365 PMC7453721

[28] Jarrín OF, Nyandege AN, Grafova IB, Dong X, Lin H. Validity of race and ethnicity codes in Medicare administrative data compared with gold-standard self-reported race collected during routine home health care visits. Med Care. 2020;58(1):e1-e8. doi:10.1097/MLR.0000000000001216 31688554 PMC6904433

