# Error in Funding/Support Information

**DOI:** 10.1001/jamanetworkopen.2023.27045

**Published:** 2023-07-20

In the Original Investigation titled “Trends in the Use of Gabapentinoids and Opioids in the Postoperative Period Among Older Adults,”^1^ published June 16, 2023, certain aspects of James D. Harrison’s funding information were incorrect. This article has been corrected.
