# Supplementary material for: Trends in the Use of Gabapentinoids and Opioids in the Postoperative Period Among Older Adults
Source: JAMA Netw Open. 2023 Jun 16;6(6):e2318626. doi: 10.1001/jamanetworkopen.2023.18626 (PMC10276300; doi:10.1001/jamanetworkopen.2023.18626)
Supplement: Supplement 1. — eTable 1. Included Surgical Procedures and Groupings eTable 2. List of Outpatient Procedures Using HCPCS or CPT codes and Inpatient Procedures Using ICD-9-CM or ICD-10-PCS Codes eFigure. Flowchart Inclusion Criteria eMethods 1. STROBE and RECORD Guideline Responses eMethods 2. Surgical Procedures Explanation eMethods 3. List of Opioids Used in Analysis [file jamanetwopen-e2318626-s001.pdf]

Supplementary Online Content

Bongiovanni T, Gan S, Finlayson E, et al. Trends in the use of gabapentinoids and opioids in the postoperative period among older adults. *JAMA Netw Open*. 2023;6(6):e2318626. doi:10.1001/jamanetworkopen.2023.18626

eTable 1. Included Surgical Procedures and Groupings

eTable 2. List of Outpatient Procedures Using *HCPCS* or *CPT* codes and Inpatient Procedures Using *ICD-9-CM* or *ICD-10-PCS* Codes

eFigure. Flowchart Inclusion Criteria

eMethods 1. STROBE and RECORD Guideline Responses

eMethods 2. Surgical Procedures Explanation

eMethods 3. List of Opioids Used in Analysis

This supplementary material has been provided by the authors to give readers additional information about their work.

© 2023 Bongiovanni T et al. *JAMA Network Open*.

eTable 1. Included Surgical Procedures and Groupings

Total Shoulder Arthroplasty	Orthopedic
Total Hip Arthroplasty	Orthopedic
Prostatectomy, Laparoscopic	Laparoscopic
Hysterectomy, Laparoscopic	Laparoscopic
Cholecystectomy, Laparoscopic	Laparoscopic
Initial Inguinal Hernia Repair, Open	Open Abdominal
Initial Inguinal Hernia Repair, Laparoscopic	Laparoscopic
Lumbar Laminotomy	Spine
Lumbar Laminectomy	Spine
Hysterectomy, Vaginal	Open Abdominal
Carotid Endarterectomy	Vascular
Total Knee Arthroplasty	Orthopedic
Low Anterior Resection, Laparoscopic	Laparoscopic
Ventral Hernia Repair, Open	Open Abdominal

© 2023 Bongiovanni T et al. *JAMA Network Open*.

eTable 2. List of Outpatient Procedures Using *HCPCS* or *CPT* codes and Inpatient Procedures Using *ICD-9-CM* or *ICD-10-PCS* Codes

Procedure	CPT code	ICD9/10
Total Shoulder Arthroplasty	23472	81.51, 81.54, 81.88, 81.80, 81.81, 81.83, 81.23
Total Hip Arthroplasty	27130 , 27134	81.21, 81.4, 81.51
Prostatectomy, Laparoscopic	55866	60.2*, 60.3*, 60.4*, 60.5* 60.6*
Hysterectomy, Laparoscopic	58571	68.3*, 68.4*, 68.5*, 68.6*, 68.7*, 68.9*
Cholecystectomy, Laparoscopic	47562	51.23, 51.24
Initial Inguinal Hernia Repair, Open	49505	550*, 53.0*, 53.1*
Lumbar Laminotomy/Laminectomy	63030, 63047	03.0*, 03.4*, 03.5*, 03.6*
Hysterectomy, Vaginal	58262	
Carotid Endarterectomy	35301	38.02, 38.12, 38.32, 38.42, 38.62
Knee Arthroplasty	27447 , 27487	81.22, 81.43, 81.47, 81.54, 81.55
Low Anterior Resection, Laparoscopic	44207	48.0*, 48.1*, 48.4*, 48.5*, 48.6*
Initial Inguinal Hernia Repair, Laparoscopic	49650	17.1*, 17.2*
Ventral Hernia Repair, Open	49560	53.41, 53.49, 53.5*, 53.61, 53.69

© 2023 Bongiovanni T et al. *JAMA Network Open*.

eFigure. Flowchart Inclusion Criteria

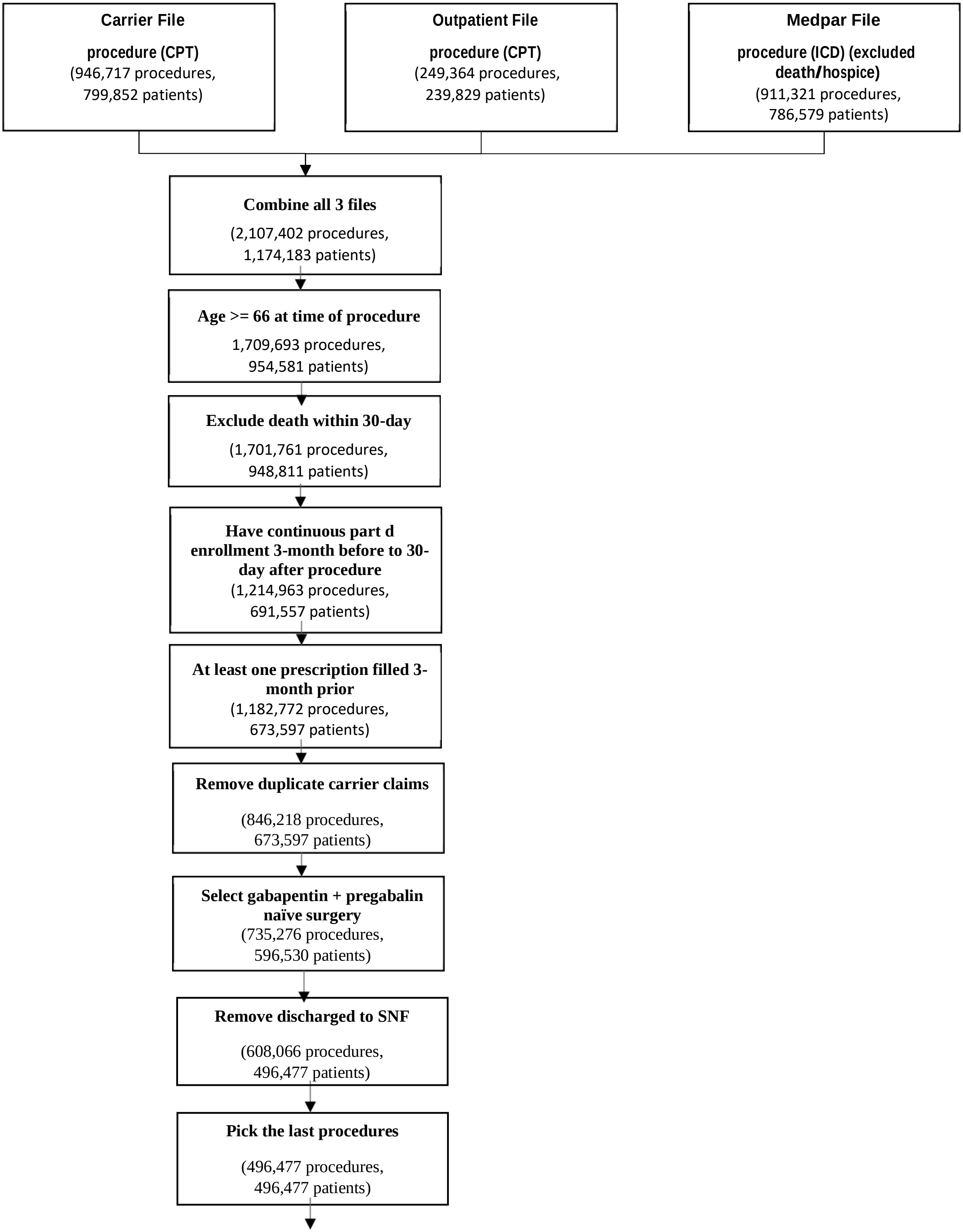

© 2023 Bongiovanni T et al. *JAMA Network Open*.

Remove multiple unrelated procedure on same date

(494,922 procedures, 494,922 patients)

© 2023 Bongiovanni T et al. *JAMA Network Open*.

eMethods 1. STROBE and RECORD Guideline Responses

RECORD 7.1: A complete list of codes and algorithms used to classify exposures, outcomes, confounders, and effect modifiers should be provided. If these cannot be reported, an explanation should be provided.

We used the HCPCS/CPT codes and ICD9-CM or ICD10-PCS codes to define the procedures (eTable2). We identified medications using the generic name of the medications (eAppendix2) in Medispan and linked that with Part D using the NCD number. We used the Medicare RTI variable for race/ethnicity. We have created a table below to link each variable with the file name and algorithm used.

Variable	Medicare File: Variable Name	Algorithm
Age	MBSF file: BENE_BIRTH_DT	Procedure Date - BENE_BIRTH_DT
Sex	MBSF file: SEX_IDENT_CD	0=Unknown<br>1=Male<br>2=Female
Race and Ethnicity	MBSF file: RTI_RACE_CD	White = 1 NON-HISPANIC WHITE;<br>Black = 2 BLACK (OR AFRICAN-AMERICAN)<br>Hispanic = 5 HISPANIC<br>Other = 0 UNKNOWN or 3 OTHER or 4 ASIAN/PACIFIC ISLANDER or 6 AMERICAN INDIAN / ALASKA NATIVE
Charlson Score	MedPAR file: DGNS_XX_CD<br>Outpatient file: ICD_DGNS_CDXX<br>Carrier file: ICD_DGNS_CDXX	Charlson score was calculated by identifying comorbidities using ICD codes from Medpar, Outpaient, Carrier claims 1 year before procedure and using algorithm from paper:<br><br>*Deyo RA, Cherkin DC, Ciol MA. Adapting a clinical comorbidity index for use with ICD-9-CM administrative databases. Journal of Clinical Epidemiology 1992;45(6):613-619.*
Gabapentinoid days supply at discharge	Part D file: DAYS_SUPLY_NUM	Gabapentinoid days supply was calculated by adding up DAYS_SUPLY_NUM variable from gabapentin or pregabalin prescription claims between 7 days before and 7 days after procedure. If gabapentin days supply is not missing, then used gabapentin days supply for Gabapentinoid days supply. If gabapentin days supply is missing, then used pregabalin days supply for Gabapentinoid days supply.
Facility type	Carrier file: LINE_PLACE_OF_SRVC_CD	Inpatient= MedPar claims or Carrier claim with LINE_PLACE_OF_SRVC_CD=21<br>Oupatient=Outpatient claims or other Carrier claims
Inpatient length of stay	MedPAR file: ADMSN_DT, DSCHRG_DT	DSCHRG_DT- ADMSN_DT
Surgery planned	MedPAR file: ER_CHRG_AMT<br>Outpatient file: REV_CNTR<br>Carrier file: LINE_PLACE_OF_SRVC_CD	Emergency surgery= MedPAR file: ER_CHRG_AMT > $0 or Outpatient file: REV_CNTR 0450-0459, 0981 or Carrier file: LINE_PLACE_OF_SRVC_CD 20 or 23

© 2023 Bongiovanni T et al. *JAMA Network Open*.

Care complexity	MedPAR file: ORG_NPI_NUM<br>Outpatient file: OP_PHYSN_NPI<br>Carrier file: PRF_PHYSN_NPI	Care complexity was calculated by counting numbers of different NPI 6 months before procedure
Type of procedure	MedPAR file: SRGCL_PRCDR_XX_CD<br>Outpatient file: HCPCS_CD<br>Carrier file: HCPCS_CD	Procedure type was identified using HCPCS/CPT codes, and ICD9-CM or ICD10-PCS codes in eTable 2.

RECORD 12.1: Authors should describe the extent to which the investigators had access to the database population used to create the study population.

Siqi Gan and Dr John Boscardin had full access to all the data in the study and takes responsibility for the integrity of the data and the accuracy of the data analysis.

RECORD 12.2: Authors should provide information on the data cleaning methods used in the study

The Medicare data was 'clean', however we created a clean data cohort which we describe in eFigure 1. Please see the variable list above for RECORD 7.1 to better understand how each variable was created.

RECORD 12.3: State whether the study included person-level, institutional-level, or other data linkage across two or more databases. The methods of linkage and methods of linkage quality evaluation should be provided.

We used Medicare 20% Data which is person-level data. We linked data as described in the methods section in the manuscript. The data was linked over time by the beneficiary ID as provided by Medicare/ResDAC. By using the 20% data, we are able to link all patients and the linkage quality is evaluated by Medicare/ResDAC prior to the data being sent to us for use.

RECORD 22.1: Authors should provide information on how to access any supplemental information such as the study protocol, raw data, or programming code.

Our study protocol is defined in the methods section of the manuscript. To access raw data, a data use agreement with Medicare and ResDac is required, and that can be requested here: https://resdac.org/. Access to the data needs to be directly through Medicare and cannot be accessed through our study team. An IRB is required. Access to programming code will be considered by request, please contact the corresponding author directly.

© 2023 Bongiovanni T et al. *JAMA Network Open*.

eMethods 2. Surgical Procedures Explanation

We selected these procedures, using prior literature, with the aim of including common procedures in older adults, but also with the understanding that these procedures would be reasonably expected to cause some amount of pain (for example, reference 17 includes cataract surgery, which we did not include as this is not a surgical procedure found to have the need for postoperative opioids). We further refined our list after study of the cohort revealed that in fact older adults often have two procedures on the same day, or that procedures greatly overlap (example; laminotomy and laminectomy) and it would not make sense to exclude these double procedures since they were so common, therefore we created these procedures as stand-alone procedures in our cohort.

eMethods 3. List of Opioids Used in Analysis

Butorphanol
Codeine
Dihydrocodeine
Fentanyl
Hydrocodone
Hydromorphone
Meperidine
Morphine
Oxycodone
Oxymorphone
Pentazocine
Tapentadol
Tramadol

© 2023 Bongiovanni T et al. *JAMA Network Open*.
