# Supplementary material for: Trends in the Use of Gabapentinoids and Opioids in the Postoperative Period Among Older Adults
Source: JAMA Netw Open. 2023 Jun 16;6(6):e2318626. doi: 10.1001/jamanetworkopen.2023.18626 (PMC10276300; doi:10.1001/jamanetworkopen.2023.18626)
Supplement: Supplement 2. — Data Sharing Statement [file jamanetwopen-e2318626-s002.pdf]

Data Sharing Statement

Bongiovanni. Trends in the Use of Gabapentinoids and Opioids in the Postoperative Period Among Older Adults. *JAMA Netw Open*. Published June 16, 2023.
doi:10.1001/jamanetworkopen.2023.18626

Data
Data available: No

Additional Information
Explanation for why data not available: These data are used through a data use agreement with Medicare and therefore cannot be shared; researchers who want access to these data should contact Medicare directly.
